# Suppression of Notch1 and AKT mediated epithelial to mesenchymal transition by Verrucarin J in metastatic colon cancer

**DOI:** 10.1038/s41419-018-0810-8

**Published:** 2018-07-23

**Authors:** Deeksha Pal, Ashish Tyagi, Balaji Chandrasekaran, Houda Alattasi, Murali K. Ankem, Arun K. Sharma, Chendil Damodaran

**Affiliations:** 1 Department of Urology, University of Louisville, Louisville, KY USA; 2 Department of Pharmacology, Penn State Cancer Institute, Penn State College of Medicine, Hershey, PA 17033 USA; 3 Department of Pathology, University of Louisville, Louisville, KY USA

## Abstract

Epithelial to mesenchymal transition (EMT) in colorectal cancer (CRC) has been attributed to activation of AKT and Notch1 signaling pathways. As EMT corresponds to increased aggressiveness of CRC, approaches that prevent metastasis by targeting AKT/Notch1 pathways are at the forefront of current research paradigms. This study examined the anti-metastatic potential of Verrucarin J (VJ), a small molecule, in CRC cells overexpressing AKT and Notch1. VJ significantly inhibited AKT/HCT 116 cell growth by acting on the AKT/NFκB/Bcl-2 signaling axis and initiated apoptotic signaling as was evident from increased expression of pro-apoptotic markers such as cleaved PARP, cleaved caspase 3, and cleaved caspase 9. Also, VJ inhibited the cell growth in AKT/Notch1-overexpressing CRC cells and abrogated EMT. The down-regulation of AKT and Notch1 signaling was apparent in immunoblot analysis and corresponded with down-regulation of mesenchymal markers including Snail, and β-catenin. Intraperitoneal administration of VJ in control (pCMV/HCT 116) and AKT/HCT 116 mice significantly suppressed AKT-induced tumor growth in a xenograft model. In addition, down-regulation of prosurvival markers as well as AKT and Notch1 was observed in the immunohistochemical analysis of the xenografted tumors. In conclusion, our study substantiates the role of AKT and Notch1 in cell proliferation, angiogenesis, and EMT of CRC cells and demonstrates that VJ may be a viable therapeutic option to counter AKT-induced cell proliferation and tumor outgrowth in CRC.

## Introduction

Distant metastasis is the manifestation of tumor invasion and is often the final and fatal step in the metastatic cascade of carcinomas. The prerequisites for tumor invasion are the acute changes in cellular attributes such as adhesion^1^. Changes in the migratory and adhesion potential of cells enable tumor cells to dissociate and migrate from the primary tumor site. These changes are characteristic of an important developmental process, which is termed as epithelial-to-mesenchymal transition (EMT)^2^. The down-regulation of epithelial cell surface markers such as β-catenin and the nuclear translocation of transcription factors such as Snail are some of the representative indicators of EMT^3^.

Recent studies have shown that hyperactivation of AKT signaling pathway plays pivotal role in metastatic cancers^4–6^. These studies have specifically demonstrated that AKT regulates EMT-specific markers to induce EMT of human squamous carcinoma cells^5^. However, metastasis is a complex phenomenon and several interrelated yet independent signaling pathways are implicated in its occurrence. Notch1 signaling is one such pathway, upstream of AKT signaling, and has been shown to modulate EMT in several cancer types including colorectal adenocarcinoma^7,8^. Crosstalk between Notch1 and Nuclear factor-κB (NFκB- p65) leads to activation of transcription factors involved in prosurvival signaling of cancer cells and contributes to colorectal cancer (CRC) cell proliferation and tumorigenesis^4^. NFκB is a family of transcription factors that plays an essential role in cancer initiation, survival, and progression. Notch1 activation induces phosphorylation of Iκβα, and activation of NFκB is mediated by kappaβ kinase^5,4^.

CRC is the third leading cause of cancer-related deaths in the United States^9^, despite dramatic reductions in CRC incidence and morbidity over the past few decades^6^. Metastatic CRC, particularly, has limited treatment options and thus high mortality rates^10,11^. Therefore, targeting EMT is a mainstay of approaches conceived to counter cancer progression, especially in patients already diagnosed with high-grade polyps or localized colon cancer^12,13^.

The serine/threonine kinase AKT, also known as protein kinase B, is crucial for cell survival^14^. A hyperactivated AKT signaling pathway has been frequently observed in metastatic colon cancer^15–17^. Resistance to chemo/radiotherapy has also been attributed to AKT activation^18,19^. Targeting components of the AKT signaling pathway has been shown to effectively inhibit CRC cell outgrowth^20^. However, several other signaling pathways are active and contribute to the induction of EMT in CRC. Slug, a mesenchymal progression marker, is another direct downstream target of Notch1 signaling^21^. A positive correlation between Notch1 and Slug expression has been demonstrated to elicit EMT during tumor progression by repressing E-cadherin expression in several cancer types^22^. Both Notch and Slug are shown to induce Bcl-2 gene expression, a negative regulator of apoptosis^23^. AKT has also been demonstrated to inhibit apoptosis by phosphorylation of pro-apoptotic genes such as Bax, BAD, and procaspase 9^24^.

Therapies simultaneously targeting different but interrelated signaling cascades have shown potent inhibitory effects in human pancreatic and colon cancer cells^25–27^. More specifically, progress in CRC treatment has led to the development of small-molecule inhibitors of target proteins, including miRNAs involved in proliferation, apoptosis, and angiogenesis^28–30^. Our lab has identified a small molecule, Verrucarin J (VJ), a macrocyclic trichothecene, which is a sesquiterpenoid metabolite largely produced by fungi and species of the plant genus Baccharis, family Asteraceae. Their antiviral, anticancer, antimalarial, and antifungal roles have been described in different studies^31^. Verrucarin A, another trichothecene, is well studied in prostate, pancreatic, and breast cancer^32–34^. However, VJ, an equally potent compound, is largely neglected.

Our study indicates that VJ effectively suppresses AKT-induced tumor growth and inhibits Notch1 and AKT-mediated EMT in colon cancer. The ability to target the multifaceted functions of Notch1, AKT, their crosstalk, and interactions with various signaling intermediates in CRC with a single small molecule presents a promising approach for treatment of advanced CRC.

## Materials and methods

### Cell culture and reagents

Human colorectal cancer cell lines HCT 116 and SW-620 were purchased from ATCC (American type culture collection; Manassas, VA, USA). HCT 116 and SW-620 were maintained in McCoy’s medium and Dulbecco's modified Eagle's medium respectively supplemented with 10% fetal bovine serum and penicillin (100 units/ml) and streptomycin (100 units/ml; Millipore Sigma, St. Louis, USA) in the presence of 5% CO_2_ at 37 °C. AKT-overexpressing HCT 116 clones were grown in G418 (300 μg/ml) selection media under the conditions mentioned above.

### Cell proliferation and colony formation assay

The anti-proliferative effect of VJ was determined by the MTT ((3-[4, 5-dimethylthiazol-2-yl]-2, 5-diphenyltetrazolium bromide) assay. HCT 116, SW-620, pCMV/HCT 116, and AKT/HCT 116 were treated with varying concentrations of VJ (0.1 −0.5 μM). VJ was reconstituted in dimethyl sulfoxide (DMSO) at 10 mM stock and stored at −80 °C until further use. Inhibitory effects of VJ on cell proliferation were established using the BrdU (5-bromo-2'-deoxyuridine) incorporation assay. Anchorage-independent growth was determined by CytoSelect™ 96-well In Vitro Tumor Sensitivity Assay kit (Cell Biolabs, Inc., San Diego, CA, USA).

### Detection of apoptosis by flow cytometry

Apoptosis was detected by flow cytometry analysis of Annexin V–fluorescein isothiocyanate (FITC) against propidium iodide (PI) assay (BD Pharminogen™ FITC Annexin V Apoptosis Detection Kit I, BD Pharminogen, San Diego, CA, USA). Briefly, adherent cells were harvested after 24 h of treatment and resuspended in the Annexin V Binding Buffer (1 × 10^6^ cells/ml). Thereafter, cells were incubated with Annexin V–FITC and PI for 15 min at room temperature in the dark and immediately analyzed by flow cytometry (BD FACSCalibur™). The data is presented as bi-parametric dot plots of Annexin V–FITC against PI.

### Cell invasion

The invasive behavior of HCT 116, SW-620, pCMV/HCT 116, and AKT/HCT 116 was evaluated in Boyden chambers equipped with polyethylene terephthalate membranes with 8 μm pores. Approximately 5 × 10^4^ cells per chamber were resuspended in culture medium with vehicle (DMSO) or VJ and layered on the Matrigel (Corning BioCoat™ Matrigel Invasion Chamber, Bedford, MA, USA). After an additional 24 h, the invading cells were counted using an AMG EVOS digital inverted microscope (Life Technologies, Carlsbad, CA, USA) as previously described [35].

### Cell migration

HCT 116, SW-620, pCMV/HCT 116, and AKT/HCT 116 cells were grown to confluency in 12-well plates. A scratch through the culture was created with a pipette tip on the base of the plate. Images were immediately recorded from each well and again 24 h after wound generation. The distance that cells migrated through the scratched area was determined by NIS-Element AR software (Nikon Instruments Inc, Melville, NY, USA) and the distance between the opposing edges of the wound was measured in micrometers.

### Protein extraction and western blotting

Total protein extracts from HCT 116, SW-620, pCMV/HCT 116, and AKT/HCT 116 cells were prepared with the Mammalian Protein Extraction Reagent (Thermo Scientific, Rockford, IL, USA) according to the manufacturer’s instructions. Western blotting was performed using specific antibodies against Snail, β-catenin, Vimentin, NFκB (p65), and actin (Santa Cruz Biotechnologies, Dallas, TX, USA). AKT, pAKT, Notch1, and Bcl-2 were purchased from Cell Signaling (Danvers, MA, USA). Presnillin-1 was purchased from Genescript (Piscataway, NJ, USA). The positive bands were detected using enhanced chemiluminescence.

### Xenograft studies

BALB/c athymic nude mice (nu/nu) were purchased from the Jackson Laboratory (Bar Harbor, ME, USA) and used at 6–8 weeks of age. Animals were housed in pathogen-free conditions. To study the in vivo efficacy of Vercurin J, pCMV/HCT 116 or AKT/HCT 116 cells (1 × 10^6^) were injected subcutaneously into separate flanks of the mice (6–8 animals per group). The mice were monitored daily for tumor growth and tumor volumes were measured twice per week. VJ (0.5 mg/kg body weight) and vehicle (DMSO) was administered intraperitonealy twice per week for 4 weeks, once the tumor size was >50 mm^2^.

### Immunohistochemical analysis of xenografts

Immunohistochemistry was performed on the tumor samples from the pCMV/HCT 116 and AKT/HCT 116 xenografts. Tumors were fixed and stained with hematoxylin and eosin for pathological examination. Tumor tissue slides were dewaxed and rehydrated before antigen retrieval. They were then incubated with primary antibodies against Ki67, phosphorylated AKT, p65, and Notch1 followed by incubation with respective horseradish peroxidase-conjugated secondary antibodies at room temperature for 1 h. Diaminobenzidine (DAB Substrate Kit, Vector Laboratories, Vernon Hills, IL, USA) was used for coloration, and a dark brown color was considered to be positive staining. The Ki76 score was essentially defined as the percentage of total number of tumor cells with nuclear staining and tumors are classified as low, intermediate, and highly proliferating^35^.

### Statistical analysis

The data were presented as the mean ± standard deviation (SD or SEM). The significance of the differences between the groups was determined using the unpaired Student’s *t*-test. The significant differences were established at *p* < 0.05. All of the statistical analyses were performed using Prism 6 software (GraphPad Software Inc., La Jolla, CA, USA).

## Results

### Prosurvival signaling blockage by VJ suppresses CRC cell’s viability

**Fig. 1:**
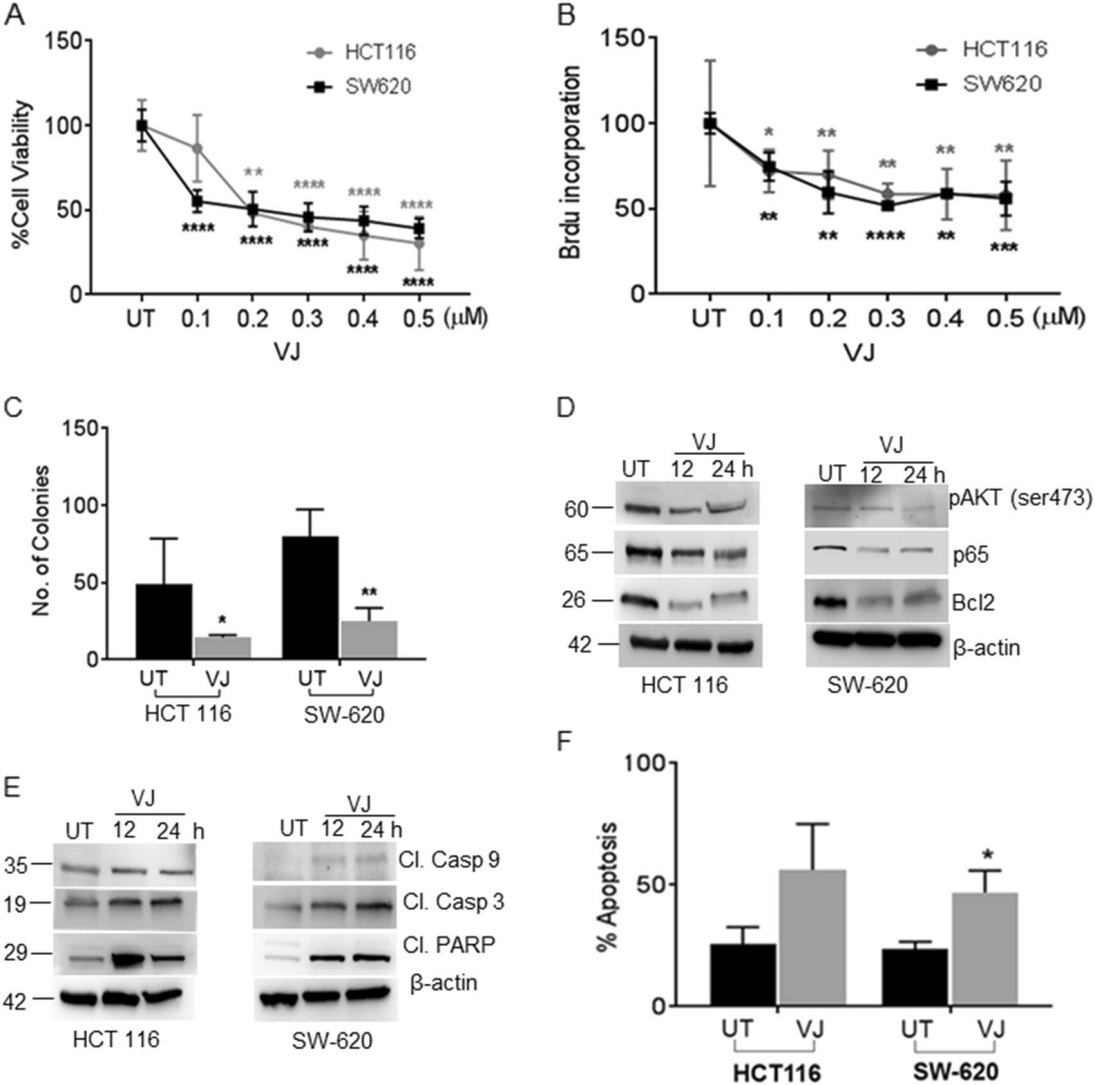
VJ inhibits cell growth, inactivates AKT kinase, and induces apoptosis in HCT 116 and SW-620 colon cancer cell lines. **a** HCT 116 and SW-620 cells were treated with indicated concentration of VJ or vehicle (DMSO) for 24 h followed by MTT assay for cell viability. **b** Cell proliferation was assessed by BrdU incorporation in HCT 116 and SW-620 cells treated with the indicated concentrations of VJ. **c** Anchorage-independent growth was assessed by a soft agar colony formation assay in HCT 116 and SW-620 cells. **d** VJ-treated cells show down-regulation of the expression of pAKT (Ser473), p65, and Bcl-2 in a time-dependent manner. **e** VJ-treated cells show up-regulation in the expression of cleaved caspase 9, cleaved caspase 3, and cleaved PARP in a time-dependent manner. **f** Apoptosis was quantified using flow cytometry of Annexin V–FITC and PI-stained, VJ-treated HCT 116 and SW-620 cells. Data are presented as the mean ± standard deviation (SEM/SD) of three independent experiments. Student’s *t*-test was used to calculate statistical significance between vehicle and treatment at each concentration; *p < 0.05, **p < 0.01 and ***p < 0.001

To determine the therapeutic potency of VJ on CRC, we examined cell viability and proliferation of CRC cells (HCT116 and SW-620) using MTT and BrdU assays. Significant reductions in cell viability was observed in both HCT 116 (50% inhibition at 200 nm; *p* = 0.0078) and SW-620 (50% inhibition at 180 nm; *p* = 0.0008) cell lines (Fig. 1a). Cell proliferation was also inhibited by about 50% in both HCT 116 cells (*p* = 0.0031) and SW-620 cells (*p* = 0.0020) when treated with 300 nm concentration of VJ (Fig. 1b).

Anchorage-independent cell growth analysis revealed the reduced colony forming ability of both HCT 116 (*p* = 0.0215) and SW-620 (*p* = 0.0088) cells treated with half-maximal inhibitory concentration (IC_50_) of VJ for 24 h (Fig. 1c). When HCT 116 and SW-620 cells were treated with VJ, significant reductions in AKT and p65 phosphorylation and Bcl-2 expression were observed (Fig. 1d). Induction of apoptosis was suggested by increased expression of cleaved caspase 3, 9, and cleaved poly (ADP-ribose) polymerase (PARP; Fig. 1e). These results were corroborated with Annexin V–FITC staining which showed significant increases in apoptosis in both cell lines (HCT 116: 30.5%, *p* = 0.0382 and SW-620: 23.2%, *p* = 0.0131) after treatment of VJ (Fig. 1f). Overall, these results suggest that VJ suppresses prosurvival signaling by inhibiting AKT activation, resulting in induction of apoptosis, which resulted in growth inhibition of CRC cells.

### VJ inhibits AKT-induced cell proliferation

**Fig. 2:**
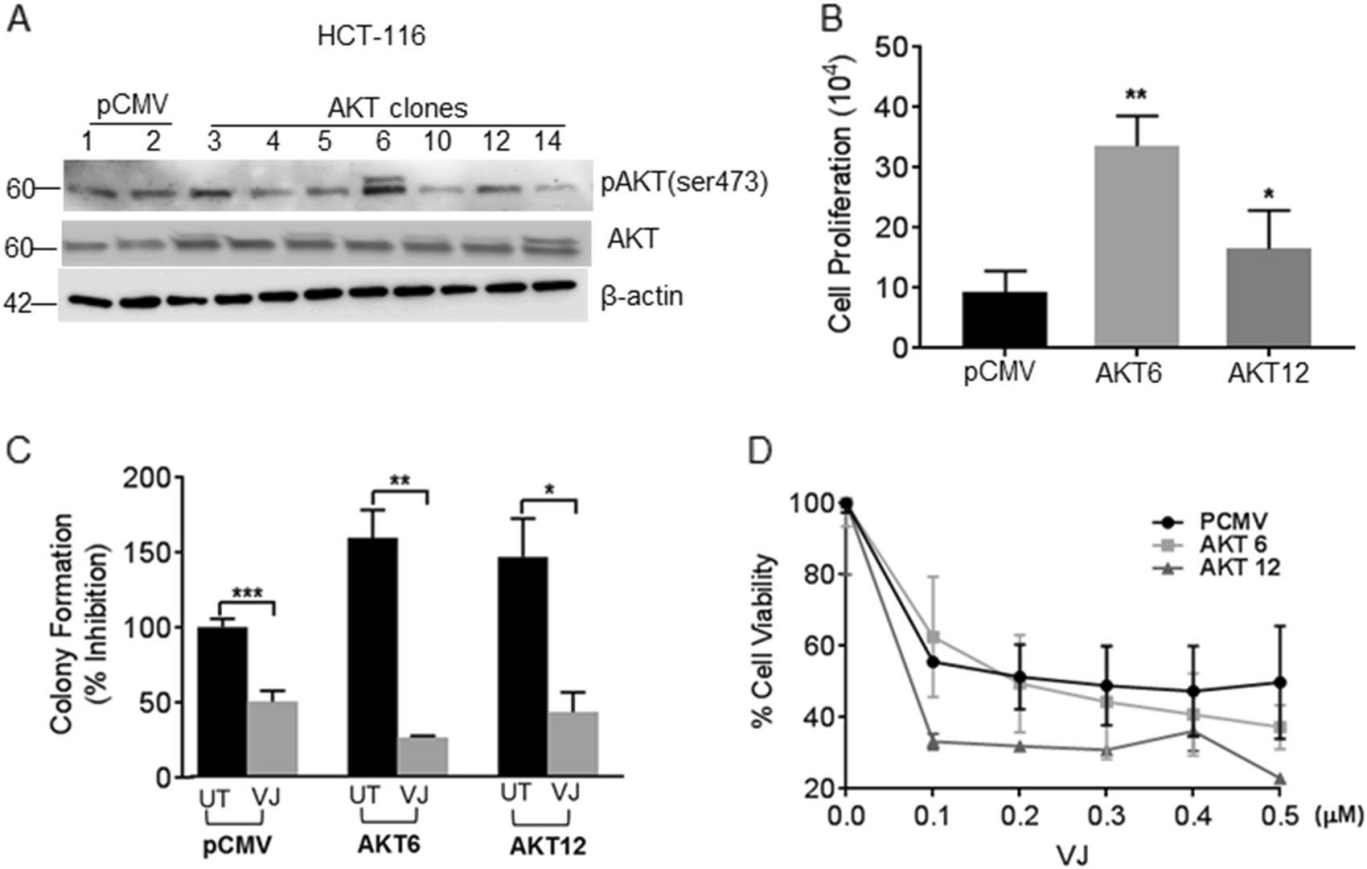
VJ suppresses cell growth in AKT-overexpressing HCT 116 cells. **a** Western blot analysis of basal expression of AKT and pAKT (Ser473) in HCT 116 (pCMV and AKT) cells. **b** BrdU incorporation assay demonstrating cell proliferation of HCT 116 stable transfectants (AKT-6 and AKT-12). **c** Colony forming assay on HCT 116 stable transfectants. **d** HCT 116 stable transfectants were treated with indicated concentration of VJ or Vehicle (DMSO) for 24 h followed by MTT assay for cell viability. Student’s *t*-test was used to calculate statistical significance between vehicle and treatment at each concentration; *p < 0.05, **p < 0.01 and ***p < 0.001

To confirm the possible role of AKT in CRC cell proliferation, we generated HCT 116 cell lines stably overexpressing AKT (AKT/HCT 116) (Fig. 2a). AKT-6 and AKT-12 were chosen for further studies based on their high AKT expression levels among the seven clones. To assess the effects on proliferation, the pCMV-transfected (control) and AKT-overexpressing clones AKT-6 and AKT-12 were incubated with BrdU. AKT-6/HCT 116 had 3.59-fold higher (*p* = 0.0418) and AKT-12/HCT 116 demonstrated 1.76-fold (*p* = 0.0030) more BrdU incorporation than the pCMV transfected HCT 116 cells (Fig. 2b). Colony forming ability was also increased in both AKT-6/HCT 116 (59.5%, *p* = 0.0161) and AKT-12/HCT 116 (46.8%, *p* = 0.0449) cells compared to pCMV transfected HCT 116 controls (Fig. 2c). Interestingly, VJ treatment (200 nm) considerably reduced the colony forming ability of pCMV/HCT 116 (49.8%, *p* = 0.0008), and a 26.6% reduction in AKT-6/HCT 116 (*p* = 0.0036) and 43.4% inhibition in AKT-12/HCT 116 (*p* = 0.0471) cells (Fig. 2c) was observed. Treatment with VJ (200 nm) resulted in an approximate 50% decrease in cell viability in both pCMV/HCT 116 and AKT-6/HCT 116 cells at 200 nm concentration, and a 50% decrease in cell viability in AKT-12/HCT 116 cells was observed at 60 nm when analyzed with MTT assay (Fig. 2d). Collectively, these results suggest that VJ can overcome AKT-induced cell proliferation and colony formation in CRC cells.

### Suppression of AKT-mediated survival induces apoptosis in AKT/HCT 116 cells

**Fig. 3:**
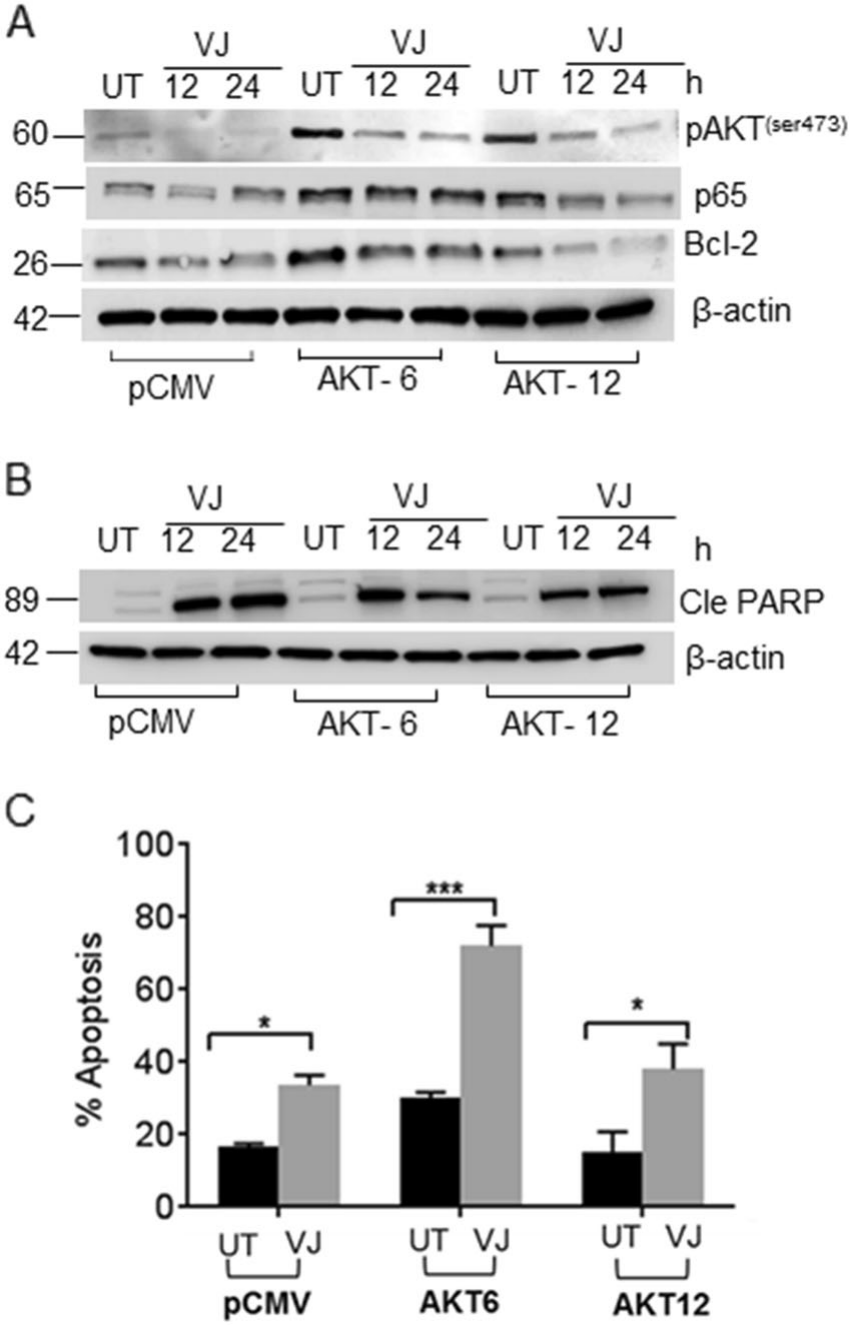
Inhibition of AKT signaling and induction of apoptosis by VJ in HCT transfectants. **a** Total protein lysates from VJ-treated pCMV/HCT 116 and AKT/HCT 116 cells were analyzed for prosurvival markers pAKT, NFκB (p65), and Bcl-2. **b** Total protein lysates from VJ-treated pCMV/HCT 116 and AKT/HCT 116 cells were analyzed for pro-apoptotic marker cleaved PARP expression. **c** Apoptotic assays were performed using Annexin V–FITC and PI staining in non-transfected and AKT-overexpressing HCT 116 transfectants treated with the indicated concentration of VJ or vehicle (DMSO). Student’s *t*-test was used to calculate statistical significance between vehicle and treatment at each concentration; *p < 0.05 and ***p < 0.001

Next we examined whether VJ blocks AKT-mediated signaling in AKT-overexpressing HCT 116 cells. VJ inhibited the expression of pAKT (Ser473) in all HCT 116 transfectants (PCMV/HCT 116 and AKT-6/HCT 116; AKT-12/HCT 116) (Fig. 3a). Additionally, a concomitant decrease was seen in downstream prosurvival proteins p65 and Bcl-2, which had higher expression levels in the AKT-overexpressing clones (Fig. 3a). These results explain the observed up-regulation of pro-apoptotic marker cleaved PARP (Fig. 3b), and the induction of apoptosis in VJ-treated pCMV/HCT 116 (16.9%, *p* = 0.0108) as well as VJ-treated AKT-overexpressing AKT-6/HCT 116 (42%, *p* = 0.0003) and AKT-12/HCT 116 cells (23%, *p* = 0.0125) was seen by FACS analysis (Fig. 3c).

### VJ inhibits Notch1-mediated cell growth

**Fig. 4:**
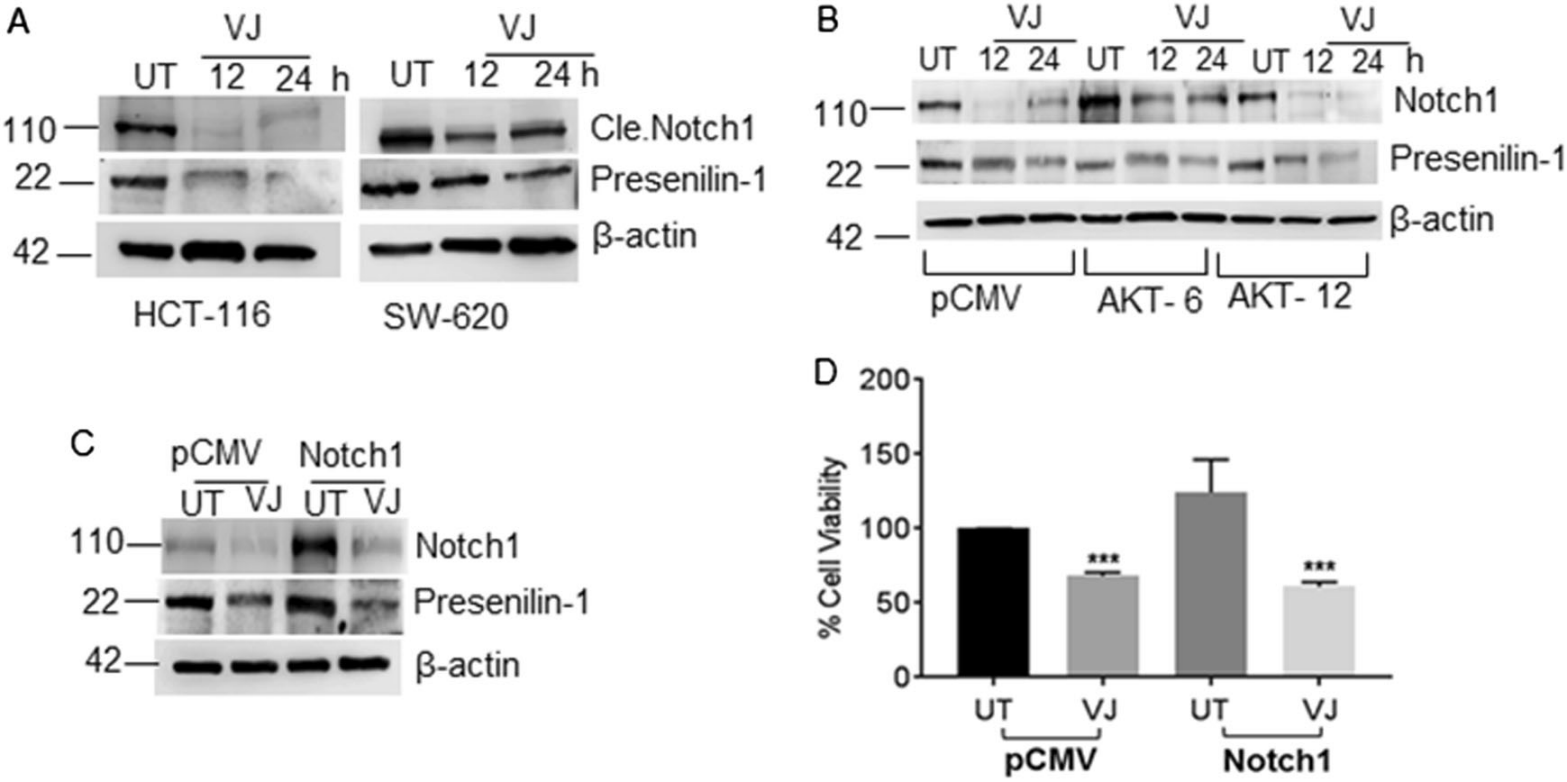
VJ down-regulates Notch1 in HCT 116 transfectants. **a** Western blot analysis of VJ-treated HCT 116 and SW-620 cells for Notch1 and Presenilin 1 expression. **b**, **c** Immunoblot analysis for the expression of Notch1 and Presenilin 1 in AKT/HCT 116 stable clones and Notch/HCT 116 cells. **d** Stable clones from HCT 116 (pCMV and Notch1) were treated with the indicated concentration of VJ or vehicle (DMSO) for 24 h followed by the MTT assay. Student’s *t*-test was used to calculate statistical significance between vehicle and treatment at each concentration; ***p < 0.001

AKT hyperactivation and Notch1 up-regulation are both regulated through NFκB activity^36^. In immunoblotting analysis, we observed elevated NFκB expression in AKT-overexpressing clones (Fig. 3a). To further analyze the influence on Notch1 expression, we established that the basal expression levels of Notch1 in HCT 116 cells were reduced by VJ (200 nm, 24 h) treatment (Fig. 4a). An apparent impact of AKT overexpression on Notch1 was observed as immunoblotting analysis revealed that Notch1 expression levels were higher in both AKT-overexpressing clones (Fig. 4b). Interestingly, Notch1 expression was down-regulated along with its downstream component presenillin 1 in VJ-treated AKT-overexpressing clones (Fig. 4b). This observation prompted us to generate Notch1/HCT 116 clones that stably overexpress Notch1 to enable further understanding of the possible role of Notch1 in CRC cell growth and VJ efficacy. We observed that VJ treatment also reduced Notch1 expression in Notch1-overexpressing Notch1/HCT 116 cells (Fig. 4c). We found a 24.4% increase in the growth of Notch1/HCT 116 cells compared to pCMV-transfected controls (Fig. 4d). This cell growth was significantly hampered by VJ treatment in both control (*p* = 0.0060) and Notch1/HCT 116 cells (*p* = 0.0008) (Fig. 4d).

### VJ overcomes AKT-induced EMT in CRC cells

**Fig. 5:**
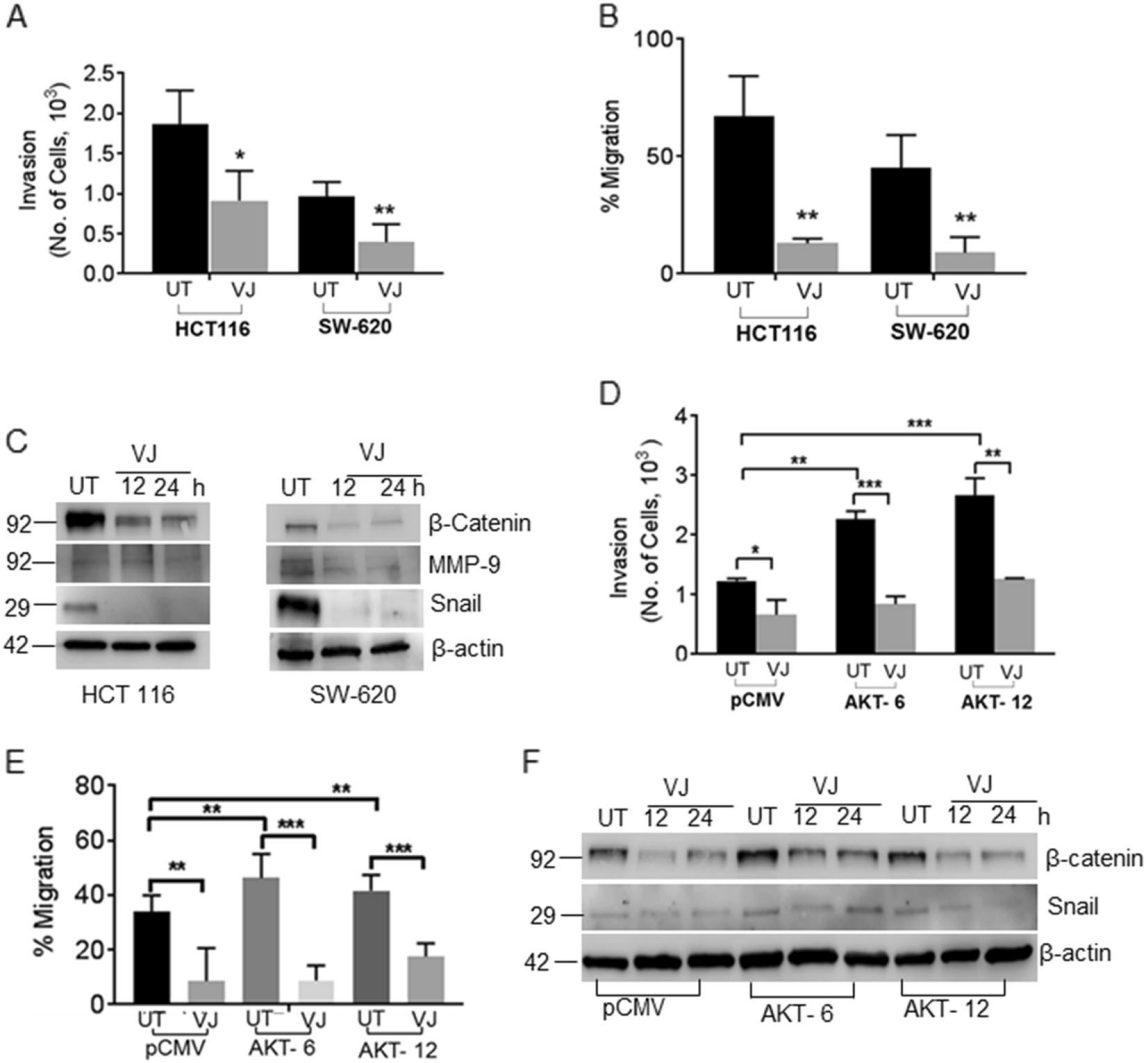
VJ inhibits EMT in AKT-overexpressing HCT 116 stable transfectants. **a** A transwell invasion assay was performed using Boyden chambers. The invaded cells were stained with crystal violet and counted. **b** HCT 116 and SW-620 cells were treated with VJ, and a linear wound across the monolayer was created. The wound gap width was measured (mm) using the Image J software. **c** VJ-treated and vehicle-treated HCT 116 and SW620 cell lysates were subjected to western blot analysis using β-catenin, MMP-9, and Snail antibodies. **d** Boyden chamber assay was used to assesses transwell invasion. Non-transfected pCMV/HCT 116 and AKT-overexpressing AKT/HCT 116 transfectants were treated with vehicle or VJ, then invaded cells were stained with crystal violet and counted. **d** pCMV/HCT 116 and AKT/HCT 116 cells were treated with VJ, and a linear wound across the monolayer was created. The wound gap width was measured (mm) using the Image J software. **f** The non-transfected pCMV/HCT 116 cells and AKT-overexpressing AKT/HCT 116 transfectants were treated with vehicle alone (DMSO) or VJ and cell lysates were subjected to western blot analysis with β-catenin and Presenilin 1 antibodies. Student’s *t*-test was used to calculate statistical significance between vehicle and treatment at each concentration; *p < 0.05, **p < 0.01 and ***p < 0.001

We next aimed to delineate the role of this VJ-induced AKT/Notch1 down-regulation in EMT. EMT-related markers were analyzed in VJ-treated HCT 116 and SW-620 cells by immunoblotting. Results indicated that VJ significantly reduced invasive (*p* = 0.0364, Fig. 5a) as well as migratory (*p* = 0 .0040, Fig. 5b) potential and down-regulated EMT markers such as β-catenin, Snail, and matrix metallopeptidase-9 (MMP-9), which are hallmarks of EMT and downstream of AKT/Notch1 (Fig. 5c). The increased invasive (Fig. 5d) as well as migratory (Fig. 5e) ability of AKT-6/HCT 116 (*p* = 0.0041; *p* = 0.0052 respectively) and AKT-12/HCT 116 (*p* = 0.0008 and *p* = 0.0212 respectively) compared to non-transfected cells were also observed. Next, to examine whether VJ can overcome aberrant expression of AKT in CRC cells, we treated HCT transfectants (pCMV/HCT 116 and AKT-6/HCT 116; AKT-12/HCT 116 clones) with VJ. As expected, VJ significantly inhibited the invasive (Fig. 5d) and migratory potential (Fig. 5e) of AKT-6/HCT 116 (*p* = 0.0004) and AKT-12/HCT 116 (*p* = 0.0012) cells. Furthermore, this reduction in the invasiveness of CRC cells also corresponded with significant decreases in the expression of EMT markers including β-catenin and Snail, as revealed in the immunoblot analysis (Fig. 5f).

### VJ reduces AKT-mediated tumor burden in xenografts

**Fig. 6:**
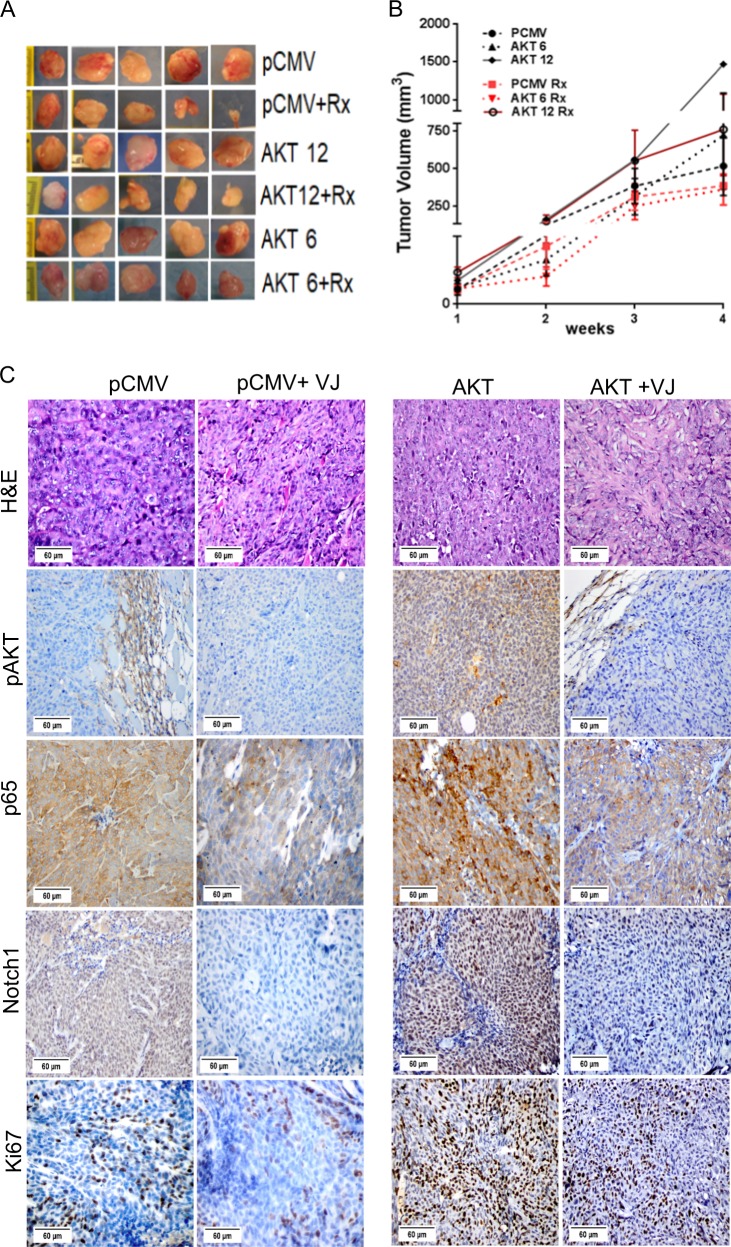
AKT and Notch1 inhibition by VJ reduces tumor burden in AKT-overexpressing in vivo model. **a** Tumors dissected and assessed from mouse xenografts of pCMV/HCT 116 or AKT/HCT 116 cells. **b** Tumor volumes were measured once per week for 4 weeks, and a line graph was plotted to compare tumor growth and volume (mm^3^) for VJ –treated pCMV/HCT 116 and AKT/HCT 116 tumors. **c** Tumor sections from AKT-overexpressing tumors and pCMV/HCT 116 tumors stained immunohistochemically with pAKT, NFκB (p65), Notch1, and Ki67

We have published that xenografts with AKT-overexpressing cells are highly aggressive compared to non-transfected pCMV/HCT 116 xenografts^37^ and that plant-derived compounds (e.g., Withaferin A) significantly inhibit this tumor growth^38^. To confirm our current in vitro results in a similar in vivo model, pCMV/HCT 116 and AKT/HCT 116 cells were injected into mice. As predicted by our earlier studies, tumors from animals injected with the AKT/HCT 116 cells grew more rapidly and aggressively, as is evident from the higher tumor volume of AKT-6/HCT 116 and AKT-12/HCT 116 injected mice than pCMV/HCT 116 tumors (Fig. 6a). An analysis of tumor volumes following VJ administration for 4 weeks revealed that VJ inhibited the growth of pCMV/HCT 116, AKT-6/HCT 116, and AKT-12/HCT 116 tumors (Fig. 6a, b) as compared to tumors in animals administered with only vehicle (PBS+DMSO).

### VJ delays AKT-induced tumor growth by AKT/Notch1 inhibition

In order to assess whether proliferation and/or survival are affected by AKT inhibition in vivo, we stained the tumors with Ki67, pAKT, p65, and Notch1. Immunohistochemical analyses of the tumors originating from pCMV/HCT 116 and AKT/HCT 116 xenografts revealed significantly more proliferating (i.e., Ki67-positive) cells in the latter. These tumors also contained high numbers of AKT, Notch1, p65-positive cells (Fig. 6c) compared to PCMV/HCT 116 tumors (Fig. 6c). Similar to the in vitro results, VJ treatment significantly reduced proliferation (Ki67 labeling index) in tumors originating from the pCMV transfected HCT 116 cells as well as in those from the AKT-overexpressing cells (Fig. 6c). In addition, VJ treatment significantly reduced the expression of prosurvival markers pAKT, Notch1, and p65 in all tumors (Fig. 6c). These findings suggest that the observed inhibition of cell and tumor growth was dependent on dampened AKT and Notch1 signaling by VJ.

## Discussion

In the present study, we demonstrated that VJ, a natural small molecule, can overcome the aberrant activation of the AKT and Notch1 signaling pathways, resulting in significant inhibition of proliferation and EMT in both in vitro and in vivo CRC models without inducing noticeable toxicity. The AKT and Notch1 pathways play a pivotal role in the transduction of anti-apoptotic and metastatic transition signals in CRC^39^. Hence, pharmacological inhibition of AKT has been the focus of several recent studies. However, their success has been limited by the toxic nature of some inhibitors (e.g., LY294002, wortmannin) and the constrained clinical usage of others (e.g., rapamycin^40^, pictilisib^41^). Several small natural compounds such as brassinin^42^, resveratrol^25^, and quercetin^40^ have safely achieved AKT inhibition in cancer models including CRC, without causing the cytotoxic effects associated with the conventional therapies^43^, but their potency is still questionable as their viability is largely subject to combination with existing conventional therapies^43^. In this regard, our results are particularly relevant and promising.

Constitutive activation of AKT contributes to the etiology and progression of CRC^8^. AKT regulates cell survival^44^ and apoptotic machinery by directly and indirectly phosphorylating pro-apoptotic effectors (e.g., Bcl-2 family proteins)^45^. Several small molecules, including topotecan^46^, perifosine^47^, wortmannin^48^, and KP372-1^49^, have caused the chemoprevention of different malignancies by inducing apoptosis in cells with constitutively active AKT^50,51^. VJ treatment significantly up-regulated cleaved PARP and caspase 3, thereby sensitizing AKT towards pro-apoptotic stimuli^48^ and shutting off cell survival pathways indicated by abrogation of Bcl-2 gene expression. These events trigger apoptosis and inhibit cell growth in VJ-treated CRC cells (HCT 116 and SW-620) including HCT 116 cells overexpressing AKT in the current study.

In addition to its anti-apoptotic effects, AKT activation is also a key character in the EMT of CRC^52^. Hyperactivation of the PI3K/AKT/mTOR (phosphatidylinositol-3-kinase/AKT/mammalian target of rapamycin) signaling pathway has been correlated to radioresistance and EMT in prostate cancer^53^ and chemoresistance in many different types of metastatic cancer such as breast cancer^18,54,55^ and CRC^56^. Previous studies from our lab demonstrated that AKT activation contributes significantly to triggering EMT in CRC cells^38^. In the present study, VJ inhibited AKT activation without affecting total AKT expression. VJ also significantly reduced MMP-9 expression, a protease strongly associated with metastasis and invasion^57^, and β-catenin, a key component of the WNT signaling pathway^58^, ultimately leading to inhibition of EMT.

Notch1 signaling is another pathway implicated in EMT in several cancer types^59^. The aberrant expression of Notch1 elicits EMT and tumor progression in cancer cells through Slug, a Snail family member and a direct downstream target of Notch1^60^. Also, crosstalk between the Notch intracellular domain (NICD) and NFκBp65 in CRC is an important phenomenon responsible for the aggressive invasion of cancer cells^61^. VJ treatment resulted in attenuation of Notch1 as well as NFκB-p65 expression in CRC cells in vitro as well as xenografts in vivo. The inhibition of Notch1/NFκB-p65 signaling might be the reason for the alleviation of AKT/Notch1-induced EMT in both the in vitro and in vivo models used in the present study. The ability of VJ to overcome AKT/Notch1 overexpression and to serve as a potential inhibitor of cancer progression was evident in vivo results, wherein cell proliferation and tumor burden were significantly reduced in the AKT/HCT 116 xenografts.

In summary, here we have demonstrated that AKT/Notch1 overexpression stimulates CRC cell outgrowth and that VJ is a potent small molecule that can overcome AKT/Notch1-induced cell proliferation and EMT in CRC cells. While it remains to be confirmed whether AKT and Notch1 pathways are interrelated or independent of each other in invasive CRC, it is clear that hyperactivation of both can lead to EMT in in vitro and in vivo CRC models. Hence, a single, natural, small molecule such as VJ that simultaneously and safely targets multiple pathways represents a promising alternative to prevailing chemotherapeutics for the treatment of metastatic colon cancer.
